# Fast, visual specialization for reading in English revealed by the topography of the N170 ERP response

**DOI:** 10.1186/1744-9081-1-13

**Published:** 2005-08-09

**Authors:** Urs Maurer, Daniel Brandeis, Bruce D McCandliss

**Affiliations:** 1 Sackler Institute, Weill-Cornell Medical College, Box 140, 1300 York Ave. New York, NY, 10021, USA; 2 Department of Child and Adolescent Psychiatry, Brainmapping Research, University of Zurich, Neumunsterallee 9, CH-8032 Zurich, Switzerland

## Abstract

**Background:**

N170 effects associated with visual words may be related to perceptual expertise effects that have been demonstrated for faces and other extensively studied classes of visual stimuli. Although face and other object expertise effects are typically bilateral or right-lateralized, the spatial topography of reading-related N170 effects are often left-lateralized, providing potential insights into the unique aspects of reading-related perceptual expertise.

**Methods:**

Extending previous research in German [1], we use a high-density channel array to characterize the N170 topography for reading-related perceptual expertise in English, a language with inconsistent spelling-to-sound mapping. N170 effects related to overall reading-related expertise are defined by contrasting responses to visual words versus novel symbol strings. By contrasting each of these conditions to pseudowords, we examined how this reading-related N170 effect generalizes to well-ordered novel letter strings.

**Results:**

A sample-by-sample permutation test computed on word versus symbol ERP topographies revealed differences during two time windows corresponding to the N170 and P300 components. Topographic centroid analysis of the word and symbol N170 demonstrated significant differences in both left-right as well as inferior-superior dimensions. Words elicited larger N170 negativities than symbols at inferior occipito-temporal channels, with the maximal effect over left inferior regions often unsampled in conventional electrode montages. Further contrasts produced inferior-superior topographic effects for the pseudoword-symbol comparison and left-lateralized topographic effects for the word-pseudoword comparison.

**Conclusion:**

Fast specialized perception related to reading experience produces an N170 modulation detectable across different EEG systems and different languages. Characterization of such effects may be improved by sampling with greater spatial frequency recordings that sample inferior regions. Unlike in German, reading-related expertise effects in English produced only partial generalization in N170 responses to novel pseudowords. The topographic inferior-superior N170 differences may reflect general perceptual expertise for orthographic strings, as it was found for words and pseudowords across both languages. The topographic left-right N170 difference between words and pseudowords was only found in English, and may suggest that ambiguity in pronunciating novel pseudowords due to inconsistency in spelling-to-sound mapping influences early stages of letter string processing.

## Background

The N170 is a component of the event-related potential (ERP) peaking between 150 and 200 ms and showing an occipito-temporally negative and fronto-centrally positive topography. It is strongly elicited by certain classes of visual stimuli, such as faces [2,3], relative to other visual control stimuli. Investigations of the psychological principles that drive the N170 to respond more strongly to some classes of stimuli over others have demonstrated *perceptual expertise *effects across several classes of stimuli, including enhanced N170 responses (relative to other object control stimuli) for bird experts viewing birds [4], car experts viewing cars [5], and has even been demonstrated for laboratory-induced expertise with 3D novel figures ("greebles" [6]). These results support a potential relationship between extensive visual experience with a stimulus domain and alterations in visual processes within the first 200 ms of perceptual identification. This framework of perceptual expertise may also account for experience-dependent changes in reading skill – a domain in which extensive practice develops considerable visual expertise at the level of letter-strings and the pattern by which letters typically are combined to create visual word forms [7].

Neurophysiological studies have shown that skilled adult readers develop fast, perceptual identification processes that are specialized for words and other letter strings, reflected by differences in N170 responses compared to control stimuli, such as symbol strings, that control for visual features [1,8-10].

Unlike findings of right-lateralized or bilateral N170 responses for faces, N170 responses to word stimuli showed a left-lateralized topography [1,3,11-13]. Across studies, however, the degree of the left-lateralization varied between strong [3,12] and moderate [1,13].

Some studies also showed that the N170 is sensitive for linguistic processing [14,15]. Consonant strings had larger N170 amplitudes than words [14,15], and sublexically irregular pseudowords were in between [14]. Other studies, however, did not find N170 differences between words and pseudowords [1,8,16]. In one study, the differences between consonant strings and words were only found for lexical and semantic tasks, but not during implicit reading [8], whereas it was found across semantic, passive, and implicit viewing in another [14]. These results suggest that N170 responses are somewhat variable across experiments, which might be due to different task demands and presentation modes.

Word frequency effects in the N170 were more consistently found across studies, with low frequency words producing more negative N170 amplitudes [17-21] (but see also [22]).

One recent study of native German speaking adults demonstrated sublexical N170 effects by contrasting responses to strings of novel symbols to letters strings grouped into common orthographic patterns (familiar or novel word forms in German), demonstrating that specialization of processing as assessed by either lexical or sublexical strings is strongest at inferior occipito-temporal channels [1]. However, the region that demonstrated the peak effect was at the edge of the montage of electrodes applied, suggesting that this effect might be better characterized by an electrode array, which covers regions inferior to the classical 10–20 or 10-10 electrode montages [23,24].

**Figure 1:**
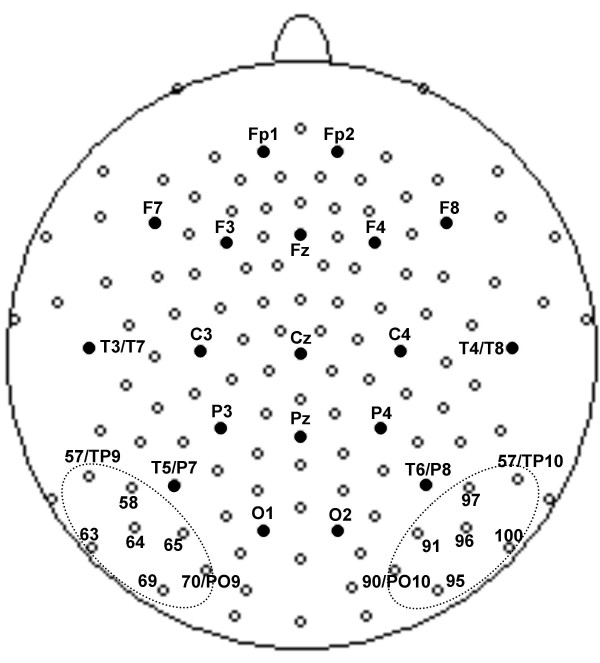
**High-density 129-channel montage. **Filled black dots indicate electrodes corresponding to the 10–20 system positions [34]. Inferior occipito-temporal channel groups used for waveform illustration and additional lateralization analyses are marked with dotted circles. Note that the high-density montage extends both the 10-10 and 10-5 montages [24] for an additional inferior row (approx. 5% of the Nasion-Inion distance).

The aim of the current study was to further characterize the nature of reading-related N170 expertise effects by applying a 129-channel array (geodesic sensor net, Electrical Geodesics, Inc.) that extends the coverage of the classical 10-10 montage [23,24] to more inferior regions, thereby providing adequate spatial sampling of the peak effect of interest (Fig. 1). This study adopts a paradigm used in a previous N170 study conducted with German speaking subjects [1] to explore potential replicability of effects across languages that differ in the level of consistency of how letters map onto word sounds [25], as well as to examine the topography of the N170 with a greater spatial sampling of inferior regions that might be critical to capturing effects produced near ventral posterior brain regions (see [7] for review). EEG was recorded continuously as participants actively monitored for an occasional target, defined as an immediately repeated item (i.e. "one-back"), among a series of word, pseudoword, or symbol string stimuli. Advantages of this paradigm include relatively equal engagement for all classes of stimuli, which can be assessed via behavioral responses to targets, as well the segregation of response-free trials from infrequent target trials within ERP analyses.

## Results

### Behavior

Participants detected targets with a high accuracy across all conditions (>90% in each condition, see table 1), indicating that all conditions were relatively easy. Subtle condition differences were detectable, however, via repeated measure ANOVA analyses, which revealed a main effect of stimulus condition for accuracy (*F*(2,13) = 6.23, *p *< 0.05), but not for reaction time (*F*(2,13) = 0.61, *p *= ns). Symbol strings were detected slightly less accurately than the other two conditions.

**Table 1:** Behavioral results for detecting targets

	**Words**	**Pseudowords**	**Symbols**
Accuracy (% correct)	98.4	95.5	90.1
Reaction time (ms)	609	599	542

### Word-symbol differences in consecutive ERP maps

**Figure 2:**
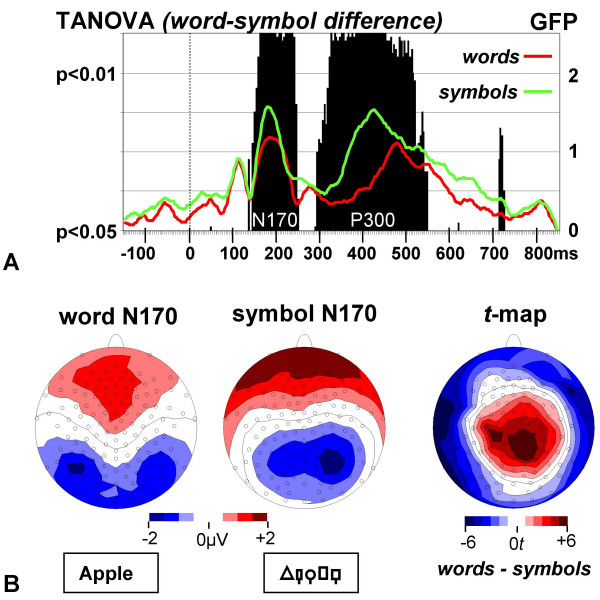
**A. Point-to-point differences (TANOVA) between word and symbol ERP maps superimposed with Global Field Power. **Significant differences (black bars) between word and symbol processing were found in two time windows corresponding to the N170 and P300 GFP components. **B. N170 maps for words and symbols and their difference *t*-map. **Words elicited larger N170 negativity than symbols at inferior occipito-temporal channels, especially over the left hemisphere. Note that the channels with the largest negative difference are located inferior to the classical 10-10 montage system.

To assess differential processing of words and symbol strings over time, a Topographic Analysis of Variance (TANOVA, [26]) on non-normalized (raw) ERP maps was computed for each time point. TANOVA on raw maps detects all systematic amplitude differences between two maps (i.e. including all 129 electrodes). Accordingly, word and symbol processing differed (*p *< 0.01, to adjust for multiple comparisons) during two separate time windows, from 160–244 ms and from 324–512 ms, largely overlapping with the Global Field Power (GFP [27]) peaks of the N170 and P300 ERP components (Fig. 2).

### N170 time window

Our approach to analyzing topographic effects in the N170 time window follows an ERP mapping approach [28,29], designed to take full advantage of information from all the channels in the high-density channel array. According to this approach, ERPs are seen as a series of maps changing in Global Field Power (GFP [27]) and topography over time. Moreover, ERP topographies tend to remain stable for short periods of time, typically changing at time points with low GFP. To get a robust measure for the N170 component, we averaged samples across the time segment between the two GFP minima (based on the average of word and symbol grandmeans) that marked the beginning and end of the N170 component as in [1]. The N170 segment maps (144–248 ms) of the word, pseudoword, and symbol conditions were subsequently analyzed to characterize GFP and topography effects across these stimulus groups. Topographic effects were tested using difference map *t*-statistics, centroid analyses, and analyses of selected channels. First, overall reading-related N170 effects were analyzed, comparing words vs. symbol strings. Second, generalization of reading-related N170 specialization to novel word forms was tested comparing pseudowords both to symbols and to words.

### Statistical difference map analyses

The word N170 topography showed the largest negativity at occipito-temporal electrodes with a maximum over the left hemisphere and the largest positivity at fronto-central electrodes. The symbol N170 topography showed the largest negativity at occipito-parietal electrodes with a maximum over the right hemisphere and the largest positivity at fronto-polar electrodes. The two topographies clearly differed, as shown by the large *t*-values in the statistical difference map (Fig. 2). The maximal effects were found at parietal electrodes and at left inferior temporal and occipito-temporal electrodes at the edge of the channel array (Fig. 2).

**Figure 3:**
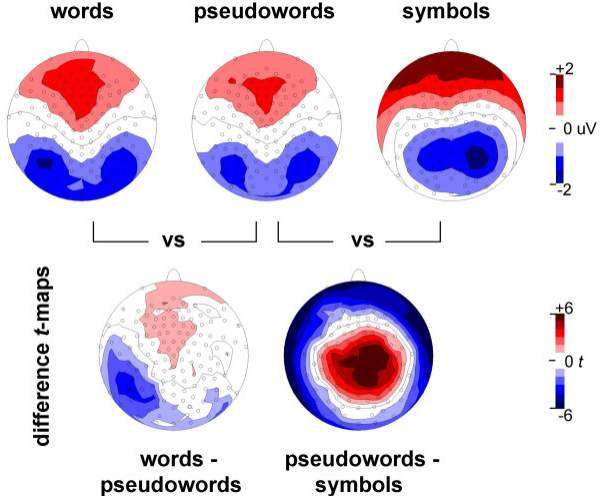
**Generalization of reading-related N170 expertise to novel word forms**. The pseudoword N170 differs from both the symbol N170 and the word N170, but the two effects show distinct topographies. The pseudoword-symbol effect shows large differences at inferior (surrounding negative difference) and superior (central positive difference) locations. The word-pseudoword effect is most pronounced over left occipito-temporal electrodes. Critical *t*-values are 2.14 (p<0.05), 2.98 (p<0.01), and 4.14 (p<0.001).

N170 responses to pseudowords and symbols clearly differed in the statistical difference map (Fig. 3). The maximal effects were found at parietal electrodes and bilaterally at inferior electrodes at the edge of the electrode array.

The statistical difference map also indicated clear N170 effects between responses to words and pseudowords (Fig. 3). These effects were left-lateralized, as they were found at many occipito-temporal channels over the left hemisphere, but hardly over the right hemisphere. The pseudoword N170 topography resembled the word N170, but showed a more bilateral negativity and a positivity centered around the fronto-central midline electrodes, in contrast to the more left-lateralized negativity and positivity of the word N170 (Fig. 3).

### Global Field Power analysis

To assess overall map strength, we ran a repeated measure Analysis of Variance (ANOVA) on the N170 GFP value separately for the overall reading-related contrast (word vs. symbol), and for the two contrasts testing generalization of reading-related N170 specialization to novel word forms (pseudowords vs. symbols and words vs. pseudowords).

There was no significant overall reading-related effect in the N170 GFP measure, although map strength was somewhat larger for symbols than for words (*F*(1,14) = 3.00, *p *= ns, see also Fig. 2). The additional comparisons revealed that N170 GFP was larger in response to symbols than to pseudowords (*F*(1,14) = 6.07, *p *< 0.05), but did not differ between words and pseudowords (*F*(1,14) = 2.13, *p *= ns), although map strength was slightly larger for words than pseudowords.

### Topographic centroid analyses

We used centroid measures (centers of gravity) of the positive and negative fields on the scalp surface to characterize the ERP topography [30-32]. The 3D locations of the positive and negative centroids were computed from all 129 electrode positions (in x-, y-, and z-Talairach space [33]) weighted by their positive or negative values, respectively. Repeated measure ANOVAs were run on the centroid positions, separately for the overall reading-related contrast (words vs. symbols), and the two contrasts testing for generalization to pseudowords (pseudowords vs. symbols and words vs. pseudowords). Positive and negative centroids were grouped in a factor "polarity", as positive and negative poles are often systematically related in ERP maps, and the three spatial coordinates were treated as multivariate dependent measures. Contrast main effects and polarity interactions are only reported if they differ significantly (*p *< 0.05) at the multivariate level. For multivariate significant effects, univariate tests were computed for the x-, y-, and z-axes, to characterize the nature of the multivariate effect in 3D space. Contrast main effects are referred to as "mean centroids" (positive and negative centroids showed a similar pattern), and contrast-by-polarity interaction effects are referred to as "centroid distribution" (positive and negative centroids showed a different pattern).

**Figure 4:**
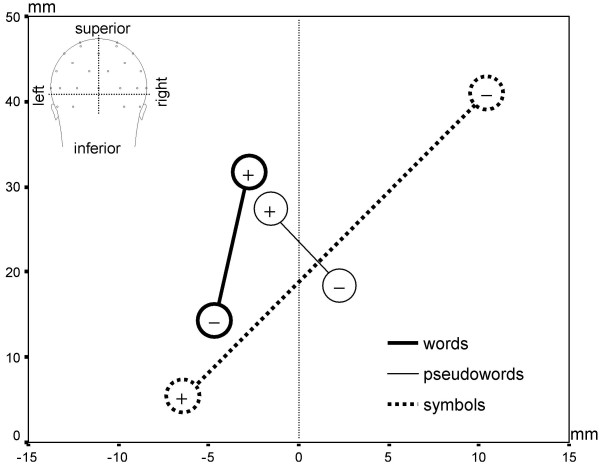
**Positive and negative centroids of the N170 word, pseudoword, and symbol topographies. **Symbol centroids show a different pattern from word and pseudoword centroids, with a reversed polarity in the inferior-superior direction. In addition, the negative centroid is left-lateralized for words, but right-lateralized for symbols. The centroids are also more left-lateralized for words than for pseudowords. Note that the centroids represent the ERP topography on the scalp surface and are by no means estimations of the underlying sources.

As summarized in Table 2, the centroid analysis revealed clear overall reading-related effects in the N170, indicated by a different centroid distribution between word and symbol maps (*contrast x polarity, F*(3,12) = 10.76, *p *< 0.01). These differences appeared in both the analysis of the inferior-superior z coordinate axis (*F*(1,14) = 37.30, *p *< 0.001) and the analysis of left-right x coordinate axis (*F*(1,14) = 7.07, *p *< 0.05, table 2). As illustrated in Fig. 4, this interaction captures differences between negative centroids appearing as inferior and left-lateralized for words, yet superior and right-lateralized for symbols. The positive centroids showed a reversed pattern, as they were located more superior for words and more inferior for symbols (Fig. 4).

**Table 2:** Effects of the N170 topographic centroid analyses.

**Topographic effects **(multivariate significant)	**x-axis**	**y-axis**	**z-axis**
*Contrast **(words vs. symbols) **x polarity (positive vs. negative)*	*p < 0.05*	*ns*	*p < 0.001*
*Contrast **(pseudowords vs. symbols) **x polarity (positive vs. negative)*	ns*	*ns*	*p < 0.001*
*Contrast **(words vs. pseudowords)***	*p < 0.05*	*ns*	*Ns*

* non-significant trend (F(1,14) = 3.73, p < 0.1)

Clear topographic effects for the N170 responses to pseudowords and symbols were also found in the centroid analysis, as indicated by different centroid distributions between pseudoword and symbol conditions (*contrast x polarity F*(3,12) = 7.10, *p *< 0.01). This difference was mainly found on the z-axis (*F*(1,14) = 20.94, *p *< 0.001, table 2). The negative centroids were located more inferior for pseudowords and more superior for symbols, whereas the positive centroids showed a reversed pattern (Fig. 4). There was an additional non-significant trend on the x-axis with the centroids more lateralized for symbols than for pseudowords (*F*(1,14) = 3.74, *p *< 0.1).

Topographic effects between the N170 in response to words and pseudowords were indicated by different mean centroid locations for words and pseudowords (*F*(3,12) = 3.59, *p *< 0.05). These effects appeared on the left-right x axis (*F*(1,14) = 4.73, *p *< 0.05, table 2). The mean centroids were more left-lateralized for words than for pseudowords (Fig. 4).

### Selected waveform analyses

In order to allow comparisons with more conventional ERP analysis approaches, we also performed an analysis on left and right inferior occipito-temporal channel groups which have been shown to be most sensitive to word-symbol differences [1]. Figure 1 illustrates the specific channels included in the left and right groups, respectively. Repeated measure ANOVAs were run with the *hemisphere *factor (left vs. right channel group) separately for the overall reading-related contrast (words vs. symbols), and the two contrasts testing for generalization to pseudowords (pseudowords vs. symbols and words vs. pseudowords).

**Figure 5:**
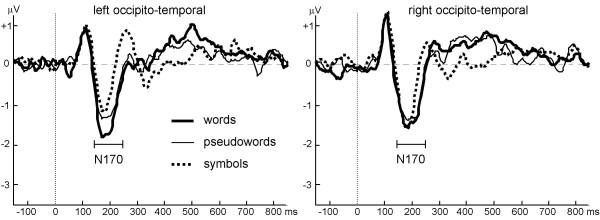
**Waveforms at left and right inferior occipito-temporal channels. **The N170 is larger for words than for pseudowords and symbols, especially at the left hemisphere channels. Pseudowords have a larger N170 than symbols at both hemispheres, especially during the late part of the N170.

A clear overall reading-related effect was seen in the selected waveform analysis comparing word and symbol N170. The N170 amplitude was larger for words than for symbols (*contrast, F*(1,14) = 9.93, *p *< 0.01), and this difference was larger over the left hemisphere *(contrast x hemisphere, F*(1,14) = 10.78, *p *< 0.01; Fig. 5).

The pseudoword-symbol contrast also revealed a significant effect. Pseudowords elicited larger N170 amplitudes than symbols (*F*(1,14) = 7.18, *p *< 0.05; Fig. 5). Although this difference was somewhat larger over the left hemisphere, the interaction with *hemisphere *failed to reach significance (*F*(1,14) = 3.26, *p *< 0.1).

The word-pseudoword contrast also revealed a significant effect in N170 amplitudes at inferior occipito-temporal channels. The amplitudes were larger for words than pseudowords (*contrast*, *F*(1,14) = 8.35, *p *< 0.05), which were more pronounced at the channels over the left than over the right hemisphere (*contrast x hemisphere*, *F*(1,14) = 7.57, *p *< 0.05; Fig. 5).

## Discussion

### Summary of the results

The present high-density ERP study clearly shows reading-related expertise effects that occur early during processing of visual words. Processing differences between words and novel symbol strings that control for basic visual features emerged in a time window corresponding to the N170 ERP component.

One of the central goals of this study involved extensive analysis of the topography of the N170 reading-related perceptual expertise effect. The N170 responses elicited by word and symbol stimulus blocks demonstrated significantly distinct topographies. The strongest topographic effect was found in a different centroid distribution on the inferior-superior coordinate axis. This effect reflected the occipito-temporal negativity and fronto-central positivity in the word maps and the occipito-parietal negativity and fronto-polar positivity in the symbol maps. This difference also led to maximal effects in the *t*-map at inferior and superior electrodes. Indeed, the largest negative effects were at the inferior edge of the channel array and might be missed by many conventional montages, which typically do not sample these regions [23,24,34]. An additional topographic effect was found in a different lateralization of the word and the symbol N170. The negative centroids were left-lateralized for words, but right-lateralized for symbols. This lateralization difference was corroborated in the selected waveform analysis, which showed larger N170 amplitudes for words than symbols especially over the left inferior occipito-temporal channels.

The results also inform the generalization of reading-related expertise in the N170 to novel word forms. The pseudoword vs. symbols N170 contrast led to large *t*-values in the statistical difference map and demonstrated strong evidence for perceptual expertise elicited by novel word forms. The topography of this N170 effect played out primarily as a shift in the inferior-superior dimension of the positive and negative centroids, very similar to the inferior-superior topographic N170 difference found between word and symbol centroids. In contrast, the topographic effect in the left-right dimension only partially generalized to pseudowords, showing just a non-significant trend for lateralization differences, which was corroborated in the selected waveform analysis.

Overall, N170 reading expertise effects do not appear to fully generalize to pseudoword probes in English, as a generalization was found for the topographic inferior-superior effect, but only partially for the topographic lateralization effect.

The strongest evidence that generalization to novel word forms differs in lateralization came from the word-pseudoword comparison, in which the N170 centroids were more left-lateralized for words than for pseudowords. This left-lateralization was corroborated in the additional analysis at inferior occipito-temporal channel groups and in the statistical difference map. This demonstrates that the left-lateralized topographic effect of reading-related visual expertise in the N170 does not generalize well to pseudowords.

Behavioral results, overall, served to ensure that the participants demonstrated roughly equivalent levels of engagement with the different classes of stimuli, although subtle behavioral differences were revealed in the case of the symbol condition, in which slightly lower accuracy suggests that detecting symbol strings was slightly more difficult compared to the other conditions. Interestingly, the equivalent speed and accuracy for target detection across words and pseudowords demonstrates that the left-lateralized word-pseudoword effect in the N170 is not dependent on processing differences assessed by behavioral measures.

### Replication of effects across languages

The present study adopted a paradigm based on an earlier study in Zurich [1] that used a different EEG system with participants speaking a different language. The paradigm was identical in the two studies, except for language-specific word and pseudoword stimuli, and the same analysis strategy was used. This allows us to examine the two sets of results regarding similarities and differences.

Overall, the basic findings regarding reading-related specialization were successfully replicated, such as major word-symbol differences during the N170 and the P300 time windows, and similar topographic N170 differences between words and symbols with larger negativities for words than for symbols at inferior occipito-temporal channels especially over the left hemisphere. Particularly, the centroid analyses of the N170 maps showed the same robust differences between words and symbols in the inferior-superior dimension in the two studies. The left-lateralized topographic effect for the word-symbol comparison in the present study also appeared in the Zurich data, where it reached significance in the last two thirds of the N170. Overall, the results suggest that word-symbol differences in the N170 time range are robust markers for rapid specialization for reading, which can be detected across different EEG systems and languages.

Although the two studies showed similar topographic N170 effects for the word-symbol comparison, reading-related specialization differed between the two languages when probed with novel word forms. For the pseudoword-symbol comparison, both studies found significant differences in the inferior-superior dimension, similar to the one for the word-symbol comparison. In the left-right dimension, however, the difference was significant in Zurich, but not in the present study. The strongest evidence for a difference in generalization to pseudowords between the two studies was found in the word-pseudoword comparison, where in contrast to the significant lateralization difference in the present study, no difference at all was found in the Zurich study. This reflects the fact that the pseudoword topographies were left-lateralized for German speakers, but bilateral in the present study. As we will discuss below, differences in orthographic depth between the two languages may explain this effect in particular and may shed some light on the characteristic left-lateralized topography of reading-related N170 specialization in general.

Behavioral results were very similar in both studies. Repetition detection was high (>90%) in all conditions, with a slight advantage for detecting words and pseudowords compared to symbols in both studies. There was no difference in reaction time between conditions in any of the two studies. This shows that the difference in generalization of reading-related expertise to pseudowords between the two studies was not due to differences in overt behavioral responses.

In addition to the generalization difference of reading-related N170 expertise to novel word forms, the two studies also differed in relative map strength of the N170 between the conditions. In the Zurich study, words and pseudowords had larger GFP than symbols, but in the present study this relation tended to be reversed. Smaller overall amplitudes in the EEG for electrolyte-net systems compared to electrogel-cap systems, as reported earlier [35], should affect language and symbol stimuli equally. Although the different word-symbol GFP ratios between the studies could potentially result from differing symbol GFP, this seems unlikely because the two studies mainly differed with respect to the language stimuli. Thus, this may suggest that words and pseudowords elicit relatively smaller N170 amplitudes in English than in German which could be due to differences in orthographic depth between the two languages. However, to exclude a possible influence of the recording system, such language-related N170 effects on GFP may be investigated in future studies using the same system for the two language groups.

In contrast, reading-related N170 effects basically replicated their general topographies across languages, which suggests that topography (rather than GFP) is a robust marker of reading-related specialization in the N170 across EEG systems and language backgrounds. This, in turn, suggests that the topographic N170 differences between the two studies found in the word-pseudoword comparisons are valid markers for the influence of different languages.

The topographic centroid analyses not only revealed similar effects for reading-related N170 specialization in the word-symbol comparison between the two studies, the centroid analysis also appeared as a more fruitful analysis strategy than the selected waveform analysis in the present study. Although the waveform analysis confirmed the lateralization effects of the centroids, the pre-selected channel array prevented the detection of the strong topographic inferior-superior effects in the word-symbol and pseudoword-symbol N170 contrasts. This illustrates, how the selection of particular channels in multi-channel recordings could lead to biased results and conclusions. The centroid analysis method is a means for unbiased topographic ERP analyses, and has been proven useful to detect topographic differences in earlier studies (e.g. [30-32]).

### General Discussion

The differences between word and symbol processing that appeared in the N170 component in the present study, corroborate findings from studies using MEG [10] or conventional EEG systems [1,8,9,36,37] in supporting the general conclusion that processes in posterior brain regions that are activated within the first 200 msec are sensitive to reading-related experience. The present study extends earlier findings in two important ways: showing that the maximal effect of the negative difference appears at scalp locations that were not sampled previously, and showing that reading-related expertise in the N170 has a distinct functional organization in English which can be detected when the expertise system is probed with pseudowords.

Reading-related expertise in the N170 appeared in two topographic dimensions: the inferior-superior dimension and the left-right dimension. These two topographic effects generalized to a different degree to pseudowords in the present study, suggesting that the two effects may be associated with different functional properties of reading-related expertise.

The inferior-superior modulation of the N170 word-symbol difference fully generalized to pseudowords in the present study. The same inferior-superior effect was also found for the word-symbol and pseudoword-symbol contrast in the Zurich study with participants speaking a different language [1]. Since the inferior-superior topographic effect is robust across languages and generalizes to novel word forms, it may reflect visual expertise for the familiarity of letters within strings. Visual expertise reflected by the inferior-superior topographic N170 effect may be related to expertise in other visual domains, such as expertise for faces or objects [2,3].

Such speculation regarding various forms of expertise, however, requires additional investigation, as studies on face or object expertise have not analyzed topographic effects beyond lateralization [4-6]. It remains to be tested whether the inferior-superior topographic effect in the N170 relates to a functional property that is shared across different domains of visual expertise, or whether it represents a functional property characteristic of reading.

Earlier findings suggested that the main characteristic feature of reading-related N170 specialization lies in its left-lateralized topography [1,3,8], contrasting the typically bilateral or right-lateralized N170 topographies for faces and objects of expertise [2,3]. The present results add further evidence for this notion, but extend earlier findings, by showing that this left-lateralized topographic effect does not fully generalize to novel word forms in English, as it did in German [1]. Thus, the left-lateralized topographic effect may be associated with functional properties that can be inferred from language differences between English and German, especially with respect to pseudoword reading.

English and German differ in the degree of orthographic depth, which is deep in English and shallow in German. Orthographic depth refers to the level of consistency whith which spelling maps onto word sounds (feedforward consistency) and word sounds map onto spelling (feedbackward consistency). In the case of pseudoword reading, the former plays an important role. Due to inconsistency in spelling-to-sound mapping in English, the pronunciation of pseudowords is much more ambiguous in English than in German. Thus the left-lateralized topographic N170 effect may specifically relate to processes involved in mapping letters onto word sounds. Mapping consistency has been demonstrated to be a central factor modulating the rise of automaticity in information processing [38]. The lack of a left-lateralization for English pseudowords may suggest that such processes are less automatic in English [39], and are engaged to a lesser degree while detecting pseudoword repetitions, because repetition detection does not require explicit pronunciation of the stimuli.

Such an interpretation also fits with the lack of N170 word-pseudoword differences in earlier studies in Finnish and French [8,16]. Whereas Finnish orthography is shallow, French orthography has some inconsistencies in sound-to-spelling mapping, but is rather consistent in spelling-to-sound mapping, which renders pseudoword pronunciation less ambiguous in French [40].

One study in English also did not find N170 differences between words and pseudowords, but the participants performed a lexical task that encouraged deeper language processing of the pseudowords, which is in agreement with the automaticity hypothesis [21].

Another study in English found larger N170 amplitudes for irregular pseudowords than for words, which may suggest that the irregularity of the pseudowords led to an enhancement of the N170 similar to findings for consonant strings [14,15]. The effect of larger N170 amplitudes for consonant strings compared to words may be related to the well-replicated findings of larger N170 amplitudes for low-frequency words than for high-frequency words [18-21]. For both the consonant strings and the word frequency effects, no lateralization differences have been reported and the inferior-superior topographic effect has not been investigated. Future studies may show whether this effect is related to the inferior-superior topographic effect in the present study, or whether it represents an additional reading-related modulation of the N170 component.

Different levels of engagement in orthographic-to-phonological processing might also explain the variable left-lateralization of N170 related to reading in the literature [3,12]. Thus, the degree of left-lateralization may vary according to language, task, and stimulus factors that impact the degree to which visual, orthographic and phonological codes are engaged.

Evidence for language-specific effects on pseudoword processing also comes from a PET study with English and Italian subjects. During explicit and implicit pseudoword processing, left posterior inferior temporal regions were more activated in English subjects, whereas in Italian subjects left superior temporal regions were more activated [25]. These results corroborate that pseudowords are processed differently in languages that differ in consistency of spelling-to-sound mapping. However, future studies combining hemodynamic and electrophysiological methods are needed to clarify the relation between metabolic activation and N170 amplitude modulation for pseudoword processing in English.

Combined hemodynamic and electrophysiological studies can also help localize the sources of the reading-related N170 specialization. Studies combining fMRI with MEG [41] and EEG [42] support the view that the word N170 originates predominantely from inferior occipito-temporal regions, in agreement with source localization from studies using MEG and EEG alone [1,3,10]. The posterior left-lateralized effect for words in the current study is consistent with sources in the left inferior occipito-temporal region, including the general region of the "Visual Word Form Area", and suggests that such a left posterior region demonstrates different patterns of neuronal responses to words vs. visual control stimuli within the first 200 msec of processing.

The notion of the Visual Word Form Area was first inspired by neuropsychological observations of "pure" alexia, or letter by letter reading, characterized by an inability to read entire words, typically following damage to left-inferior-temporal regions with a maximal probability over fusiform gyrus (see [7] for review). Left fusiform gyrus regions are also activated in metabolic studies contrasting words and visual control stimuli, and this area has been termed the Visual Word Form Area [7,42], suggesting a structure-function linkage between this region and early cognitive perceptual processes proposed in models of word recognition [38]. Although the left fusiform gyrus is typically activated in visual word tasks, this specialization does not necessarily exclude the participation of this region in other forms of processing, such as picture recognition, nor does it exclude the participation of additional regions in visual word processing (for review see [7,43]).

Previous neuroimaging studies of the putative "Visual Word Form Area" in the left fusiform gyrus have shown sensitivity for orthographic regularity, with more activation for words and pseudowords than for nonwords (for a review see [7]). In contrast, sensitivity for familiarity of word forms was small (for a review see [7]), although a more recent study suggests that activation may increase for words with low frequency and for pseudowords [44]. In the present ERP study the N170 showed sensitivity for the familiarity of word forms suggesting that this sensitivity may be language-dependent, which might also apply for the fMRI results. However, there are additional reasons that could explain different results between ERP and fMRI studies. Some N170 effects may be too transient to be captured by the low temporal resolution of fMRI. Moreover, it is also possible that the N170 effects for the word-pseudoword comparison do not originate from the left fusiform region, but from other left posterior regions contributing to the N170. Future research combining fMRI and ERP in the same study, and examining factors such as spelling-to-sound consistency patterns across languages, and within words and pseudowords, may help to elucidate the nature of different neural contributions to the N170 related to reading expertise.

## Conclusion

The present study provides further evidence that there are rapid perceptual processes in the brain that are specialized for reading. The N170 topography is a robust neurophysiological marker for this specialization showing more inferior and left-lateralized negativity for words compared to symbols. The results extend this general finding via a dense array and extended inferior coverage, demonstrating that the maximal negative effect is more inferior than reported previously. Further characterization of the N170 response in the current study suggests that reading-related perceptual expertise in the N170 can be characterized by at least two topographic effects which generalize to novel word forms to different degrees. The inferior-superior topographic effect in the N170 fully generalized to novel word forms, and may reflect expertise for letters or well-ordered letter strings. Unlike in German, the left-lateralized topographic effect in the N170 did not generalize to novel word forms in English. Inference from language differences between English and German suggests that the left-lateralized topographic effect in reading-related N170 specialization may reflect spelling-to-sound conversion, which might be less automaticly engaged in pseudoword processing in English due to more ambiguous pronunciation of novel word forms.

## Methods

### Participants

The data of 15 right-handed, native English speakers (19 to 29 years old) are presented. All subjects had normal or corrected-to-normal vision and their word reading and pseudoword decoding abilities [45] were within the normal range (within 2 SD of the norm mean). Although EEG data of 20 subjects were obtained, data of 5 subjects were discarded due to low signal-to-noise ratios (3 subjects), bad net fit (1 subject), and outlier values in the ERP (>3 SD, 1 subject). All subjects provided informed consent approved by the Weill-Cornell Institutional Review Board Committee.

### Procedure

To investigate rapid specialization for print, we used a paradigm that was used in an earlier study in Zurich, Switzerland [1]. The main differences between the two studies are the different EEG systems (geodesic net vs. electrode caps) and the language of the stimuli and participants (English vs. German). The experiment used the same stimulus string conditions (words, pseudowords, symbol strings) as [1], but while the symbol-strings were identical, the German words were translated to English (high-frequency words in both languages), and the German pseudowords were replaced by regular English pseudowords. Both words and pseudowords were printed with an initial capital letter to match the visual characteristics of German nouns. Words, pseudowords, and symbol-strings were matched for string length and contained 4.5 letters/symbols on average (range: 3–7), which also equaled the German string length. The experimental parameters were kept identical to the Zurich study: stimuli were shown every 2050 ms for 700 ms in black on a white background 100 cm away from the subject at a visual angle of 1.6–3.6 degrees (shorter distance and smaller print size to keep the same visual angle). In each condition, 72 stimuli were presented in 2 blocks containing 17% repetitions, which served as targets. To keep the experiment context the same as in the Zurich study, 2 blocks of pictures were also presented within the same session, but these data are not reported, as the different stimulus size and stimulus contrast of the pictures would confound condition effects in the N170 component. In all blocks, participants were instructed to press a button with their right thumb whenever they detected an immediate repetition.

### Electrophysiological Recording and Analyses

The 129 channel ERPs were recorded using a geodesic sensor net [46] with a Cz reference. Data were sampled at 250 Hz/channel with filter settings 0.1–100 Hz and with calibrated technical zero baselines. Impedance was kept below 50 kΩ [47]. Using BESA software, channels with excessive artifacts were spline interpolated (in average 3.5 channels per subject), and eye blinks were corrected (multiple source eye correction method [48], as applied in the Zurich study). The data then were digitally bandpass filtered (0.3–30 Hz), segmented (-150–850 ms), artifact rejected (± 100 uV), and averaged according to non-target stimuli separately for the four conditions. Using Brain Vision Analyzer software, the averaged data were re-referenced to average reference ([27]), and highpass filtered (1 Hz) to further reduce slow wave drifts. After computing Global Field Power (GFP) [27], grandmeans were computed for all four stimulus conditions.

To assess differential processing of word and symbol strings, a Topographic Analysis of Variance (TANOVA, [26], part of the LORETA-Key software package, available at  on non-normalized (raw) ERP maps was computed for each time point. TANOVA on raw maps detects all systematic amplitude differences between two maps running a nonparametric randomization test [49] on the GFP of difference maps [26,27]. Note that differences resulting from TANOVA on raw maps can be due to different topographies, as well as due to different map strengths.

For the N170 analysis, a time segment was selected between the two GFP minima before and after the N170 peak (144–248 ms) of the averaged word and symbol GFP grandmeans, as in [1]. For the N170 segment maps, GFP and 3D centroids were computed. GFP is the root mean square of the values at all electrodes. The positive 3D centroid is the voltage-weighted average of the locations of all electrodes showing positive values; the negative 3D centroid is the analogous computed for electrodes with negative values. Centroid locations are shown in Talairach space [33]. GFP and centroid measures were analyzed in repeated measure ANOVAs with a "contrast" factor (either words vs. symbols, pseudowords vs. symbols, or words vs. pseudowords). For the centroid analyses "polarity" (positive vs. negative) was included as an additional factor, and the x-, y-, and z-axes were treated as multivariate dependent measures. The selected waveform analysis in the N170 segment was computed with values from inferior occipito-temporal channel groups over the left (channels 57, 58, 63, 64, 65, 69, and 70) and right (channels 90, 91, 95, 96, 97, 100, 101) hemisphere, as inferior occipito-temporal regions were most sensitive to word-symbol differences in earlier work [1]. This analysis was similar to the GFP analysis with the additional "hemisphere" factor (left vs. right).

For the behavioral analysis two repeated measure ANOVAs were computed for accuracy and reaction time with the "condition" factor (words vs. pseudowords vs. symbols).

## List of abbreviations

3D: three-dimensional

ANOVA: Analysis of Variance

EEG: Electroencephalography

ERP: Event-related potential

GFP: Global Field Power

TANOVA: Topographic Analysis of Variance

## Authors' contributions

UM, DB, and BDM designed the study and the data analysis strategy. UM and BDM shared the elaboration of the paper. UM was responsible for data collection and data analysis.
